# Bone Morphogenic Proteins and Their Antagonists in the Lower Airways of Stable COPD Patients

**DOI:** 10.3390/biology12101304

**Published:** 2023-10-03

**Authors:** Antonino Di Stefano, Umberto Rosani, Stefano Levra, Isabella Gnemmi, Paola Brun, Mauro Maniscalco, Silvestro Ennio D’Anna, Vitina Carriero, Francesca Bertolini, Fabio L. M. Ricciardolo

**Affiliations:** 1 Divisione di Pneumologia e Laboratorio di Citoimmunopatologia dell’Apparato Cardio Respiratorio, Istituti Clinici Scientifici Maugeri, IRCCS, 28010 Veruno, Italy; isabella.gnemmi@icsmaugeri.it; 2 Department of Biology, University of Padova, Via Ugo Bassi 58/b, 35121 Padova, Italy; umberto.rosani@unipd.it; 3 Department of Clinical and Biological Sciences, University of Turin, San Luigi Gonzaga University Hospital, 10043 Orbassano, Italy; stefano.levra@unito.it (S.L.); vitina.carriero@unito.it (V.C.); francesca.bertolini@unito.it (F.B.); fabioluigimassimo.ricciardolo@unito.it (F.L.M.R.); 4 Histology Unit, Department of Molecular Medicine, University of Padova, 35121 Padova, Italy; paola.brun@unipd.it; 5 Divisione di Pneumologia, Istituti Clinici Scientifici Maugeri, IRCCS, 82037 Telese, Italy; mauro.maniscalco@icsmaugeri.it (M.M.); silvestro.danna@icsmaugeri.it (S.E.D.); 6 Institute of Translational Pharmacology, National Research Council (IFT-CNR), Section of Palermo, 90146 Palermo, Italy

**Keywords:** TGFβ, BMP4, chordin, CRIM1, BMPER, BAMBI, rehabilitation, airway inflammation

## Abstract

**Simple Summary:**

Chronic obstructive pulmonary disease (COPD) is characterized by persistent airflow limitation, usually progressive, that is associated with the development of an increased inflammatory response in the airways and lung parenchyma. Reduction in the small airways and destruction of the lung parenchyma, due to emphysema, are frequently associated with the increasing severity of the disease. Cigarette smoke is considered the major cause of COPD in Westernized countries. Strategies to identify and combat the main alterations characterizing this disease are under constant investigation. Bone morphogenic proteins (BMPs) and their antagonists are involved in the tissue development and homeostasis of various organs, including the lungs. For this reason, we studied the expression and localization of BMPs and their antagonists in the large and small airways and lung parenchyma of COPD patients, control smokers (CS), and control non-smokers (CNS). In COPD, compared to CNS, BMP4 antagonists CRIM1 and chordin were increased in the bronchial epithelium of large airways, while BMP4 was decreased. In some compartments of the peripheral lung, CRIM1 and chordin were significantly decreased compared to CNS. As testified by our in vitro experiments, the typical inflammation occurring in COPD may decrease BMP4 expression in bronchial epithelial cells; hence, treatment of these cells with BMP4 protein could reduce its proliferative capability. We also showed an imbalance between BMPs and their antagonists in the lungs of stable COPD patients. These alterations may influence the remodeling process of the diseased lung as the disease progresses.

**Abstract:**

Background: Bone morphogenic proteins (BMPs) and their antagonists are involved in the tissue development and homeostasis of various organs. Objective: To determine transcriptomic and protein expression of BMPs and their antagonists in stable COPD. Methods: We measured the expression and localization of BMPs and some relevant antagonists in bronchial biopsies of stable mild/moderate COPD (MCOPD) (n = 18), severe/very severe COPD (SCOPD) (n = 16), control smokers (CS) (n = 13), and control non-smokers (CNS) (n = 11), and in lung parenchyma of MCOPD (n = 9), CS (n = 11), and CNS (n = 9) using immunohistochemistry and transcriptome analysis, in vitro after the stimulation of the 16HBE cells. Results: In bronchial biopsies, BMP4 antagonists CRIM1 and chordin were increased in the bronchial epithelium and lamina propria of COPD patients. BMP4 expression was decreased in the bronchial epithelium of SCOPD and MCOPD compared to CNS. Lung transcriptomic data showed non-significant changes between groups. CRIM1 and chordin were significantly decreased in the alveolar macrophages and alveolar septa in COPD patients. External 16HBE treatment with BMP4 protein reduced the bronchial epithelial cell proliferation. Conclusions: These data show an imbalance between BMP proteins and their antagonists in the lungs of stable COPD. This imbalance may play a role in the remodeling of the airways, altering the regenerative–reparative responses of the diseased bronchioles and lung parenchyma.

## 1. Introduction

Chronic obstructive pulmonary disease (COPD) is characterized by persistent airflow limitation associated with the development of an increased inflammatory response in the airways and lung parenchyma. Increasing severity of the disease is associated with a reduction in the small airways and alveolar septa, mainly due to lung emphysema. Cigarette smoke is considered the major risk factor causing COPD in Westernized countries (www.goldcopd.com, access date 1 September 2023). Strategies to identify and combat the main alterations characterizing this disease are under constant investigation.

Bone morphogenic proteins (BMPs), belonging to the transforming growth factor (TGF)β family [1,2], are involved in bone and cartilage formation, organ maturation, vascular biology, inflammation, and cancer [2]. A dynamic expression of BMPs has been reported during epithelial repair [3,4,5]. BMP4 also has an anti-inflammatory action blocking nuclear factor kappa B (NF-kB) signaling [6], and treatment with BMP4 suppresses the enhanced interleukin (IL)-8 production observed following lipopolysaccharide (LPS) and/or tumor necrosis factor (TNF)α stimulation of 16HBE epithelial cells and lung macrophages [7]. 

Chordin protein is an extracellular antagonist of BMP signaling [8] but there are no studies examining its presence in COPD. Chordin-like (CHL)-1 inhibits BMP4-mediated signaling [9], binding BMP ligands and thereby reducing their functional activity [10]. Hypoxia enhances CHL-1 expression to antagonize the effect of BMP4 [11]. Treatment of a mouse model of COPD with the retinoic acid derivative Am80 resulted in alveolar recovery and was associated with increased CHL-2 expression [12].

Cysteine-rich motor neuron-1 (CRIM1) is a type-I transmembrane protein that acts as a selective antagonist of BMP4 and BMP7, reducing their secretion and expression of the mature forms [13]. CRIM1 regulates the homeostasis of endothelial cells in the coronary vasculature [14], and in A549 lung cancer cells, TGFβ1-induced CRIM1 over-expression enhanced A549 cell migration and adhesion [15].

BMP endothelial cell precursor-derived regulator (BMPER) is an extracellular modulator of BMP signaling and the BMPER/BMP ratio is critical in determining the in vivo angiogenic response [16]. BMPER levels, counteracting BMP4 expression, have been associated with endothelial cells’ angiogenic functions and anti-inflammatory effects [16,17,18,19,20].

BMPER levels are decreased in damaged epithelium whilst increased BMPER levels ameliorate epithelial homeostasis and repair [21]. 

Noggin is considered a classic BMP antagonist with high-affinity binding to BMP4 [22]. Hypoxia in mice increases BMP4 expression and selectively decreases noggin expression [23]. Noggin protein treatment normalized the excessive pulmonary arterial smooth muscle cell proliferation due to increased BMP4 levels [23]. 

We hypothesized that altered BMP expression within the bronchial mucosa and peripheral airways could be associated with the severity of COPD; therefore, we examined the expression of BMPs and their antagonists in bronchial biopsies and peripheral lung tissue of COPD patients and healthy controls using immunohistochemistry and transcriptomic analysis. We also used an in vitro epithelial cell model to examine the impact of inflammation on the release of BMPs and their antagonists and the effect of BMP4 protein treatment on bronchial epithelial cells.

## 2. Materials and Methods

### 2.1. Subjects

All COPD and healthy controls were recruited from the Respiratory Medicine Unit of the “Istituti Clinici Scientifici Maugeri” (Veruno, Italy) and the section of Clinical and Biological Sciences, University Hospital Orbassano (Turin, Italy). Archival material was used in the present study [24]. We obtained bronchial biopsies from 58 subjects for the immunohistochemical study. The characteristics of these subjects are reported in Table 1. 

Twenty-nine subjects undergoing lung resection for a solitary peripheral neoplasm were recruited for the immunohistochemical analysis of peripheral lung tissue and transcriptomic analysis of bronchial rings and lung tissue. The characteristics of these subjects are reported in Table 2. 

All COPD patients were stable, and none had been treated with theophylline, antibiotics, antioxidants, mucolytics, and/or glucocorticoids in the month prior to bronchoscopy or lung resection surgery. The study conformed to the Declaration of Helsinki; the study was approved by the Institutional Review Boards of the Istituti Clinici Scientifici Maugeri (protocol p112) and by the Ethical Committee of the San Luigi Gonzaga University Hospital (protocol n. 9544/2019).

### 2.2. Lung Function Tests and Volumes

All the subjects examined performed lung function tests with measurements of FEV_1_ and FEV_1_/FVC values. To assess the reversibility of airflow obstruction, measurements were repeated 20 min after inhalation of 0.4 mg of salbutamol in patients with FEV_1_/FVC% ≤ 70%. More details are reported in the Supplementary Materials.

### 2.3. Fiberoptic Bronchoscopy, Collection, and Processing of Bronchial Biopsies

Fiberoptic bronchoscopy was performed in all subjects reported in Table 1. Bronchial biopsies were taken from segmental and subsegmental airways. Specimens were then embedded in OCT, frozen in liquid nitrogen, and stored at −80 °C until use. Cryostat sections were obtained for immunohistochemical staining [24]. More details are reported in the Supplementary Materials. 

### 2.4. Collection and Processing of the Peripheral Lung Tissue

Bronchial rings and peripheral lung tissue were obtained from 29 subjects undergoing lung resection surgery (Table 2). Lung tissue processing was performed as previously described [24,25]. Samples were frozen in liquid nitrogen and stored at −80 °C until use. Cryostat sections were then cut for immunohistochemical staining. More details are reported in the Supplementary Materials.

### 2.5. Immunohistochemistry on OCT-Embedded Bronchial Biopsies

Sections from each sample were stained with antibodies specific for BMPs and their antagonist molecules and proteins (Table 3). 

Briefly, after blocking non-specific binding sites with serum derived from the same animal species as the secondary antibody, the primary antibody was applied at optimal dilutions in TRIS-buffered saline (0.15 M saline containing 0.05 M TRIS-hydrochloric acid at pH 7.6) and incubated for 1 h at room temperature in a humid chamber. Antibody binding was detected with secondary anti-mouse (Vector, BA 2000), anti-rabbit (Vector, BA 1000), or anti-goat (Vector, BA 5000) antibodies followed by ABC kit AP AK5000, Vectastain, and fast-red substrate (red color) or ABC kit HRP Elite, PK6100, Vectastain, and diaminobenzidine substrate (brown color). Nasal polyp sections were used as positive controls. For the negative control, normal goat (sc-2048), mouse (sc-2025), or rabbit (sc-2027) non-specific immunoglobulins (Santa Cruz Biotechnology, Santa Cruz, CA, USA) were used at the same protein concentration as the primary antibody.

### 2.6. Immunohistochemistry in Human Peripheral Lung Tissue

Immunostaining of frozen peripheral lung tissue was performed as previously described [24,25]. In the present study, frozen sections were used for immunohistochemical analysis. Endogenous peroxidase activity was blocked by incubating slides in 3% hydrogen peroxide (H_2_O_2_) in phosphate-buffered saline (PBS) followed by washing in PBS. Cell membranes were permeabilized adding 0.1% saponin to the PBS. Non-specific labeling was blocked by coating with blocking serum (5% normal goat serum) for 20 min at room temperature. After washing in PBS, the sections were incubated with the anti-BMP4, chordin, CRIM1, and BMPER primary antibodies used for bronchial biopsies (Table 3). Control slides were included in each staining run using human normal tonsils or nasal polyps as a positive control for all the immunostained sections. Slides were then incubated with chromogen-fast diaminobenzidine (DAB) as the chromogenic substance, after which they were counterstained in hematoxylin and mounted on permanent mounting medium.

### 2.7. Scoring System for Immunohistochemistry in the Bronchial Biopsies

Light-microscopic analysis was performed at a magnification of 630×. The immunostaining for all the antigens studied was scored (range: 0 = absence of immunostaining to 3 = extensive intense immunostaining) in the intact bronchial epithelium. The final result was expressed as the average of all scored fields performed in each biopsy. The intra-observer (ADS) reproducibility for the most relevant antigens studied in bronchial epithelium ranged from 1.5 to 2.3%. Immunostained cells in the bronchial lamina propria were quantified 100 µm beneath the epithelial basement membrane in several non-overlapping high-power fields until examination of the whole specimen was complete. The final result was expressed as the number of positive cells/mm^2^. 

### 2.8. Scoring System for Immunohistochemistry in the Peripheral Lung Tissue

All available bronchioles, alveolar macrophages, alveolar septa, and vessels observed in each lung tissue section were analyzed for immunopositivity. The immunopositivity was scored as 0 = absence of immunostaining, 1 = 33% of cells immunostained, 2 = 66% of cells immunostained, 3 = almost all cells positive. Intensity of immunopositivity was indicated, adding a 0.5 score point to the established score based on the number of positive cells in the bronchiolar epithelium, bronchiolar lamina propria, alveolar macrophages, alveolar septa, and lung vessels [24,25]. 

### 2.9. RNA Extraction and Sequencing from Bronchial Rings and Lung Specimens

Frozen lung parenchymal tissue was used for immunohistochemical analysis and bronchial rings from the same patients were also used for RNA extraction, sequencing, and gene expression data analysis. RNA extraction was performed with the RNAeasy micro kit (Qiagen, Hilden, Germany) following the manufacturer’s instructions. A DNA removal step was applied using 500 units of RNase-free DNase (Qiagen) at room temperature for 15 min. Total RNA was resuspended in RNase-free water (Thermo Fisher, Carlsbad, CA, USA) and the RNA/DNA concentrations in each sample were quantified using the Qubit RNA and DNA high-sensitivity Assay Kit (Thermo Fisher). RNA qualities were assessed with an Agilent Bioanalyzer 2100 equipped with an RNA nano 6000 kit (Agilent, Santa Clara, CA, USA). Due to the low RIN values obtained for the lung parenchyma samples, RNA-sequencing libraries for these samples were prepared following a 3′-end sequencing procedure using the QuantSeq 3′ mRNA-Seq Library Prep Kit FWD for Illumina (Lexogen, Vienna, Austria). Consequently, the lung parenchyma libraries were sequenced using an Illumina NextSeq500 (Cribi, UniPD, Padova, Italy) with a 75 single-end read layout. For the bronchial ring samples, no quality issues were encountered and we applied the standard Illumina library preparation procedure. Bronchial ring libraries were then sequenced with a 150 paired-end read layout (Cribi).

### 2.10. Analysis of RNA-seq Data

The raw Illumina reads were trimmed for quality using fastp [26], setting a minimal Phred quality score of 25 and removing the sequencing adaptors. Raw Illumina datasets were submitted to the NCBI Short Read Archive (SRA) under the project ID PRJNA80144. FASTQ files were imported into CLC Genomic Workbench v.21 (Qiagen, Hilden, Denmark) and analyzed to compute gene expression levels and identify differentially expressed genes (DEGs). The trimmed reads were mapped to the human reference genome (hg19, Ensembl v.99) applying the following parameters: Mismatch cost = 2; Insertion cost = 3; Deletion cost = 3; Length fraction = 0.8; Similarity fraction = 0.8; and strand-specific mapping. Expression values were counted as transcripts per million (TPMs). Baggerley’s test with false discovery rate (FDR) *p*-value correction was applied to identify DEGs, setting a cutoff of 2-fold changes (FC) and a 0.05 FDR *p*-value. Limited to the gene of interest, the expression levels were extracted from the overall dataset and further discussed. 

### 2.11. Cell Culture and Treatments

An SV40 large T antigen-transformed human bronchial epithelial (16HBE) cell line was used for the in vitro experiments [27]. Cells were cultivated until reaching 60–70% confluence. The 16HBE cells were cultured for 0–24 h. Non-treated cells were used as controls. All experiments were performed in quadruplicate for each type of treatment (BMP4 10 and 50 ng/mL, BMP4 + LDN-193189 inhibitor) and time exposure (2 h). More details are reported in the Supplementary Materials.

### 2.12. ELISA Tests in the Supernatants of Treated and Non-Treated 16HBE Cells

BMP4 (Cloud-Clone Corp. SEA014Hu, lower detection limit, 11.9 pg/mL) and chordin (Cloud-Clone Corp. SEC126Hu, lower detection limit 0.115 ng/mL) protein quantification was performed in the supernatants of GROα (R&D, 275-GR/CF, 10, and 100 ng/mL), MIP1α (Chemicon, GF010, 10 and 100 ng/mL), RANTES (Prepotech, 300-06, 10 and 100 ng/mL), IL-27 (R&D, 201-LB, 10 and 100 ng/mL) and IL-8 (Prepothec, 200-08, 10 and 100 ng/mL) protein-treated and non-treated 16HBE cells. ELISA kits were used according to the manufacturer’s instructions. 

### 2.13. BrdU Test for Cell Proliferation

A cell proliferation ELISA kit, BrdU, colorimetric kit (Merck, Rahway, NJ, USA, code 11647229001) was used to quantify bronchial epithelial cell proliferation after human recombinant BMP4 (Life Technologies, Carlsbad, CA, USA, code PHC9534) treatment (10 and 50 ng/mL) in the presence or absence of the specific BMP4 inhibitor (Merck, code SML0559-5MG, 1000 nM) [3] at 2 h after treatments.

### 2.14. Statistical Analysis Applied to Functional and Morphological Data

Functional data underwent analysis of variance (ANOVA) followed by unpaired *t*-test. Morphologic data were analyzed by the Kruskal Wallis test followed by Mann–Whitney U test. Spearman’s rank method was applied to calculate correlation coefficients. Probability values of *p* < 0.05 were considered significant. More details are reported in the Supplementary Materials.

## 3. Results

### 3.1. Clinical Characteristics of Subjects Providing Bronchial Biopsies

We studied bronchial biopsies from 18 mild/moderate (MCOPD) and 16 severe/very severe (SCOPD) stable COPD patients; and 24 controls with normal lung function, 13 of whom were current or ex-smokers, while 11 were non-smokers (Table 1). 

### 3.2. Immunohistochemistry for BMPs and BMP Antagonists in the Bronchial Epithelium of Bronchial Biopsies

No differences in the expression of BMP1, BMP2, BMP7, BMP9, BMP10, and noggin were seen in the bronchial epithelium of COPD patients compared to the non-smoking and smoking controls. The number of BMP4+ immune-stained cells was significantly lower in the bronchial epithelium of both MCOPD and SCOPD groups and in the smoking controls (CS) compared to non-smokers (CNS). 

**Figure 1:**
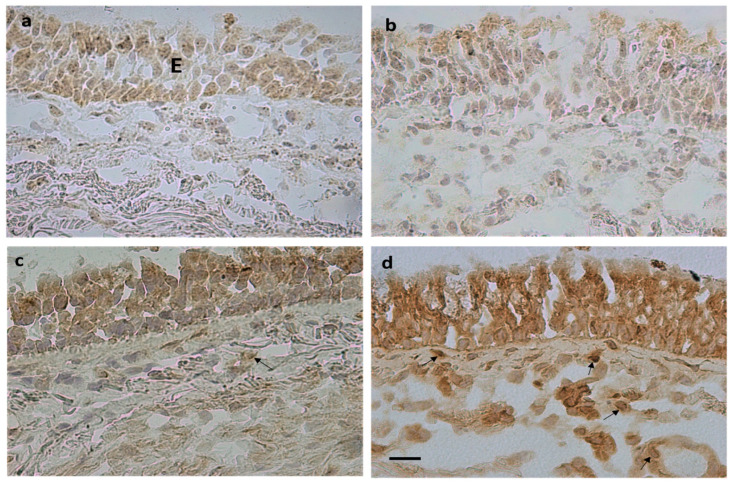
Photomicrographs showing immunostained sections obtained from bronchial biopsies of control non-smokers (**a**,**c**) and severe/very severe stable COPD (**b**,**d**). Immunostaining identifies BMP4+ cells (**a**,**b**) and chordin+ cells (**c**,**d**) in the epithelium (E) and bronchial lamina propria. Arrows (**d**) indicate immunostained endothelial cells, inflammatory cells, and fibroblasts in the lamina propria. Bar = 20 micron.

The number of BMPER+ cells was significantly increased in COPD and CS compared to CNS. Levels of CRIM1+ cells were significantly increased in both COPD groups (MCOPD and SCOPD) compared to CNS, and in MCOPD they were also increased compared to CS. Chordin+ cell numbers were significantly increased in SCOPD compared to MCOPD and CNS (Table 4, Figure 1).

### 3.3. Immunohistochemistry for BMPs and BMP Antagonists in the Bronchial Lamina Propria of Bronchial Biopsies

No differences were observed in BMP1, BMP2, BMP4, BMP7, BMP9, BMP10, BMPER, and noggin expression in the lamina propria across groups. The number of CRIM1+ immunostained cells was significantly increased in the bronchial lamina propria of MCOPD and SCOPD patients compared to CNS and CS. The number of chordin+ immunostained cells was significantly increased in SCOPD compared to MCOPD, CS, and CNS (Table 4, Figure 1). Chordin immunoreactivity was observed in endothelial cells, inflammatory cells, and fibroblasts populating the lamina propria (arrows, Figure 1).

### 3.4. Correlations between Clinical Parameters, BMPs, and BMP Antagonists in Bronchial Biopsies

**Figure 2:**
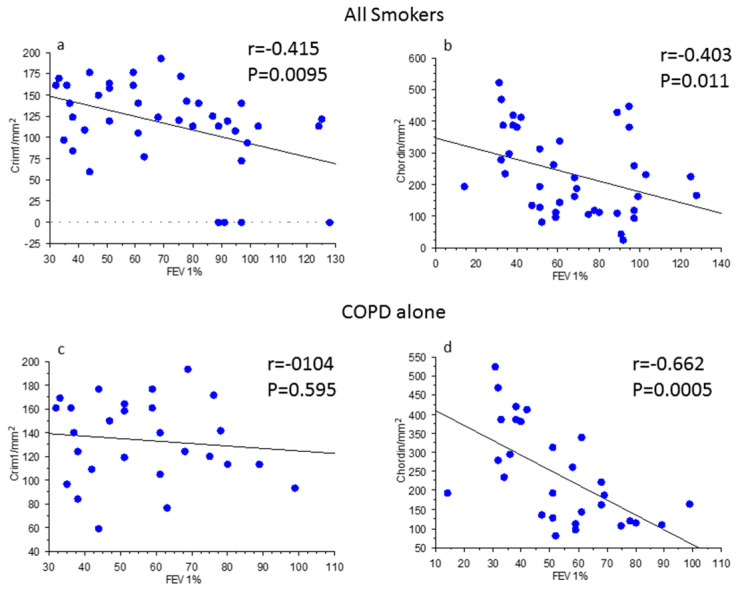
Regression analysis in all smokers (**a**,**b**) and in patients with chronic obstructive pulmonary disease (COPD) (**c**,**d**) alone. Correlations were calculated between CRIM1/mm^2^ in the lamina propria of bronchial biopsies (**a**,**c**) and forced expiratory volume in 1 s (FEV_1_) % predicted and between chordin/mm^2^ in the lamina propria of bronchial biopsies (**b**,**d**) and FEV_1_% predicted. The expression of CRIM1 and chordin in all smokers was inversely correlated with the levels of bronchial obstruction (**a**,**b**). The correlation remained significant for chordin immune expression in patients with COPD alone (**d**). Correlation coefficients were calculated using Spearman’s rank method.

When smokers with or without COPD were grouped together (Figure 2a,b) there was a significant inverse correlation between post-bronchodilator FEV_1_% predicted values and CRIM1+ cells (cells/mm^2^) (Figure 2a) and between post-bronchodilator FEV_1_% predicted values and chordin+ cells (cells/mm^2^) in the lamina propria (Figure 2b). In COPD patients alone, there was no correlation between CRIM1+ cells and post-bronchodilator FEV_1_% predicted (Figure 2c) but a significant inverse correlation remained for chordin+ cells in the lamina propria when the analysis was restricted to just patients with COPD (Figure 2d). No other significant correlations were observed between groups for all the other molecules studied.

### 3.5. ELISA Tests for BMP4 and Chordin in the Supernatants of Pro-Inflammatory Protein Treated and Non-Treated 16HBE Cells

**Figure 3:**
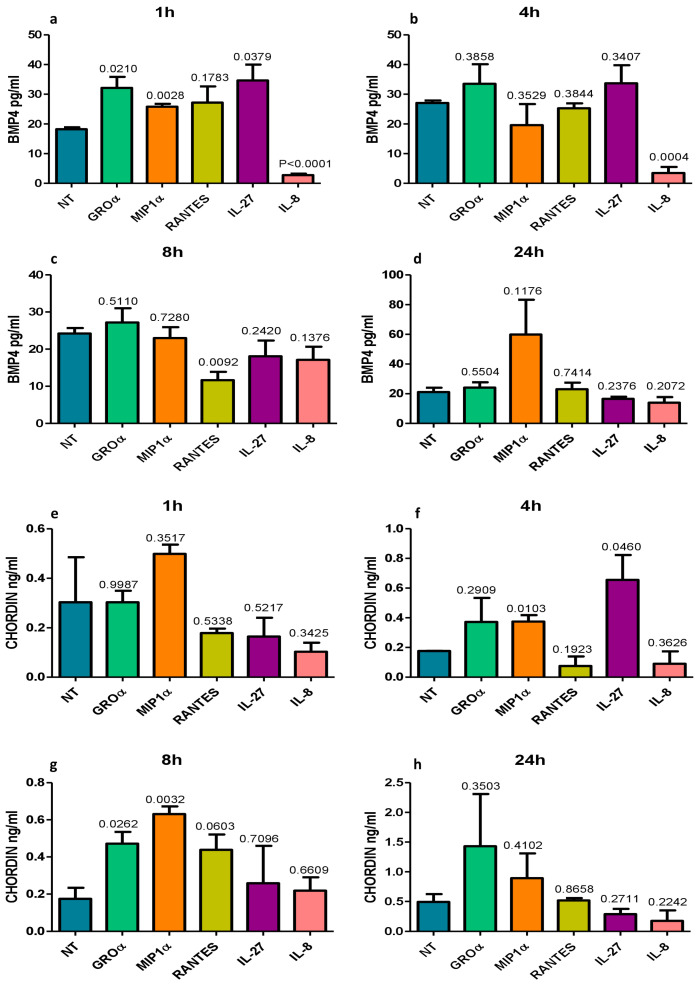
In vitro quantitation by ELISA of BMP4 (**a**–**d**) and chordin (**e**–**h**) protein secretion in the supernatants of normal primary human bronchial epithelial cells (16HBE) treated with five different pro-inflammatory molecules usually up-regulated in COPD. All challenging molecules were used at 100 and 10 ng/mL. Graphs show data obtained after the higher concentrations. RANTES significantly reduced BMP4 at 8 h (**c**) and IL-8 significantly reduced BMP4 at 1 h (**a**) and 4 h (**b**) after treatments. Chordin concentration showed a trend to increase at 8 h after RANTES (**g**) or was unchanged after IL-8 treatments (**e**–**h**). A similar trend was observed at 10 ng/mL of RANTES and IL-8 exposure. Neutrophilic inflammation may contribute to a reduction in epithelial BMP4 expression in the large airways of COPD.

The 16HBE cells were treated with the pro-neutrophilic chemokines IL-8, GROα, and RANTES and with cytokines related to monocyte–macrophage activation typically expressed in the bronchi of COPD patients (MIP1α and IL-27) [24,25] (all at 10 and 100 ng/mL) for up to 24 h (Figure 3). 

BMP4 (pg/mL) concentrations were strongly and significantly reduced by IL-8 at 1 h and 4 h after treatment and by RANTES at 8 h after treatment (Figure 3a–c). GROα, MIP1α, and IL-27 modestly but significantly increased BMP4 concentration at 1 h after treatment (Figure 3a). Chordin (ng/mL) levels were significantly increased by MIP1α and IL-27 at 4 h (Figure 3f) and by GROα and MIP1α at 8 h (Figure 3g) after treatment. IL-8 had no effect on chordin expression at any time point or concentration studied (Figure 3e–h). All treatments performed at 10 and 100 ng/mL gave similar results with a modest accentuation of differences at the higher concentration (100 ng/mL). 

### 3.6. Immunohistochemistry for BMPs and BMP Antagonists in the Peripheral Airways and Lung Parenchyma

The shortest internal diameter of the bronchioles studied, as measured in an H&E stained section from each patient was (mean ± SD) 268 ± 56, 289 ± 57, and 263 ± 81 μm, respectively, in CNS, CS, and patients with COPD. On average, the number of peripheral bronchioles studied in the three groups was 10.33 ± 5, 7.83 ± 3.8, and 5.8 ± 2.5, respectively, in CNS, CS, and patients with COPD. We examined the protein expression of chordin, CRIM1, and BMPER, the molecules showing the highest differences between groups in the analysis of bronchial biopsies. We examined bronchiolar epithelial cells, bronchiolar lamina propria, alveolar macrophages, alveolar septa, and lung vessels. BMP4 and BMPER were similarly expressed in all lung compartments studied (Table 5). 

**Figure 4:**
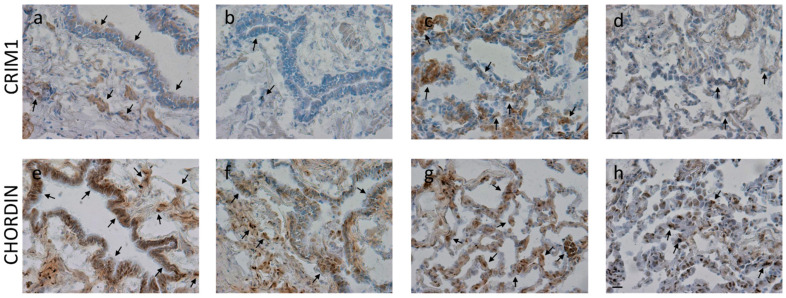
Photomicrographs showing the bronchiolar epithelium and lamina propria (**a**,**b**,**e**,**f**), alveolar macrophages, and alveolar septa (**c**,**d**,**g**,**h**) of control non-smokers (**a**,**e**,**c**,**g**) and COPD patients (**b**,**f**,**d**,**h**) immunostained for identification of CRIM1 (**a**–**d**) and chordin (**e**–**h**). Results are representative of those from 9 non-smokers and 9 mild/moderate COPD patients. Arrows indicate immunostained epithelial cells, alveolar macrophages, and alveolar septa. A reduction in immunopositivity is shown in COPD patients compared to control non-smokers. Bars = 20 microns.

Chordin levels significantly decreased in the alveolar macrophages and alveolar septa of COPD patients compared to control non-smokers. CRIM1 significantly decreased in bronchiolar lamina propria and the alveolar septa of COPD patients compared to control non-smokers (Table 5, Figure 4).

### 3.7. Gene Expression Level in Bronchial Rings and Lung Parenchyma

**Figure 5:**
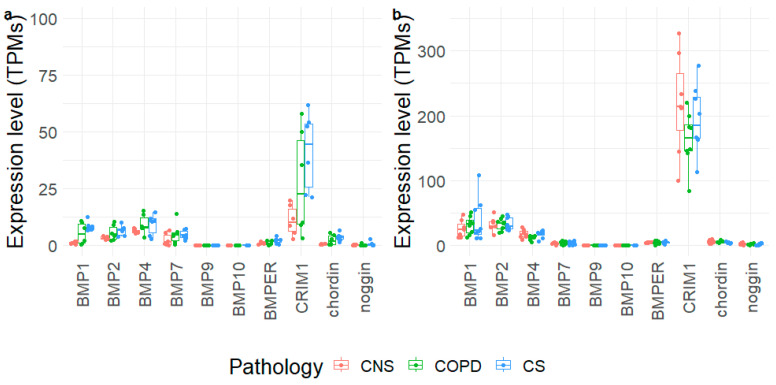
Expression levels of selected genes obtained in bronchial rings (**a**) and lung parenchyma (**b**) of control non-smokers (CNS), control smokers (CS), and patients with chronic obstructive pulmonary disease (COPD). The box plot shows the median and the distribution of expression values per gene reported as transcripts per million (TPMs).

We extracted the expression levels of BMP1, BMP2, BMP4, BMP7, BMP9, BMP10, BMPER, CRIM1, chordin, and noggin from 3′-end and whole-transcriptome data obtained from the same frozen blocks of lung parenchyma used for immunohistochemical analysis and from paired frozen bronchial rings, respectively (Figure 5). Most of the tested molecules did not show significant modifications in their mRNA expression levels across groups, and expression levels were mostly limited in the tested samples. CRIM1 showed the highest expression levels in both tissues, reaching an average of 163–217 TPMs in lung parenchyma. In bronchial rings, the expression levels of chordin were slightly lower in control non-smokers than control smokers. In lung parenchyma only, BMP1 and BMP2 expression reached considerable levels, although no significant changes were measured. Despite the lower levels of BMP1 in bronchial rings, it appeared significantly upregulated in COPD and control smoker samples compared to control non-smokers (Table S1, Figure 5). 

### 3.8. BrdU Test for Quantification of Bronchial Epithelial Cells Proliferation

**Figure 6:**
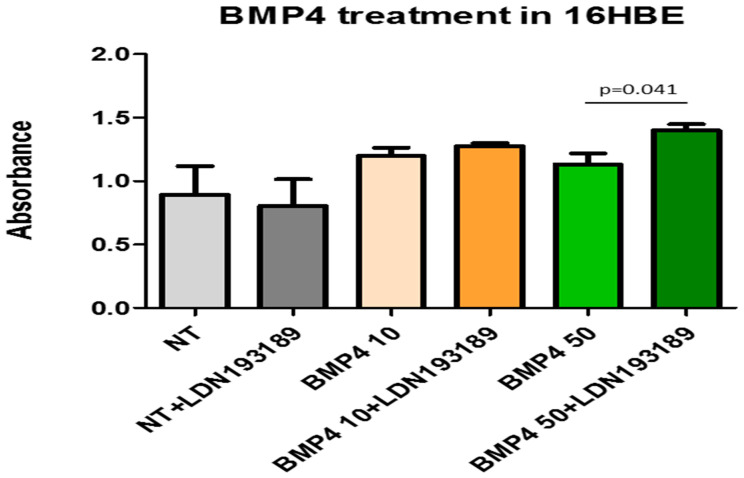
Human bronchial epithelial cells (16HBE) treated with 10 and 50 ng/mL of BMP4 alone and BMP4 + LDN193189 (BMP4 inhibitor). After 2 h of treatment, the bronchial epithelial cell profile ratio (BrDU test) was significantly higher in cells BMP4 + LDN193189 treated vs. BMP4 alone. Experiments were conducted in quadruplicate. Data expressed as mean ± SE. Statistical analysis: *t*-test. NT = non-treated cells.

Proliferation levels were measured in 16HBE cells after BMP4 treatment (10 and 50 ng/mL) in the presence or absence of a specific BMP4 inhibitor (LDN 193189, 1000 nM) at 8 h after treatment. 16HBE cells were cultivated at 9000 cells/well and after 24 h starvation were treated for a period of 8 h. Inhibition of BMP4 action at 50 ng/mL resulted in a significant increase in bronchial epithelial cell proliferation (Figure 6).

## 4. Discussion

We report here the expression and localization of BMP family proteins and BMP antagonists in bronchial biopsies of patients with stable COPD compared to control smokers and non-smokers. The expression of the BMP4 antagonists CRIM1 and chordin was increased in the bronchial epithelium and lamina propria of COPD patients. BMP4 was decreased in the bronchial epithelium of SCOPD and MCOPD compared to CNS. Chordin levels (cells/mm^2^) in bronchial biopsies were inversely correlated with levels of bronchial obstruction (FEV_1_% predicted values) both in all smokers and in patients with COPD alone. In the peripheral lung, BMP4 antagonists (chordin, CRIM1) were downregulated in alveolar macrophages and lung alveolar septa. Transcriptomic data did not show differences in mRNA expression of most BMP molecules in COPD patients compared to control groups, although they confirmed higher expression levels for CRIM1 and BMP1 compared to the other genes, whose expression was more limited. BMP1 resulted upregulated in bronchial rings of COPD and control smokers but the expression levels appeared very low. In vitro experiments indicated that the pro-neutrophilic chemokine IL-8 suppressed BMP4 expression, while derived macrophage molecules (MIP1α, IL-27) upregulated chordin release from 16HBE human bronchial epithelial cells. Inhibition of BMP4 activity in vitro upregulated bronchial epithelial cell proliferation.

A dynamic expression of BMPs, particularly BMP4, during epithelial repair has been reported in the homeostasis of the respiratory epithelium [3]. Inhibitors of BMP signaling are positive regulators of basal cell (BC) proliferation and epithelial repair, whilst exogenous BMP4 inhibits the proliferation of human nasal epithelial BCs [4]. BMPs migrate into the damaged epithelium where BMP4 downregulates E-cadherin expression and increases vimentin and αSMA expression resulting in increased cell migration [28]. This process transiently increases mesenchymal–epithelial transition (MET) formation of selective epithelial cell subtypes, which show an increased migration during wound repair until a new ‘normal’ epithelium is reconstituted [28]. Persistence of the MET phenotype in these epithelial cells may result in lung fibrosis [29]. Recently, Zuo et al. reported that high BMP4 levels inhibit basal cell proliferation and differentiation inhibiting the formation of ciliated epithelial cells and increasing squamous epithelial cell metaplasia in vitro [5].

In contrast with a previous study [5], in the large airways (bronchial biopsies) we demonstrated a decrease in BMP4 immunoreactivity in the bronchial epithelium of both SCOPD and MCOPD compared to control non-smokers. The discrepancy between our data and the previous report may reflect the more severe nature of the COPD patients in our study and the limited numbers of COPD patients studied (n = 9) by Zuo and colleagues [5]. Indeed, we found the greatest differences when comparing the SCOPD patients with CNS. We postulate that the decreased epithelial expression of BMP4 in the large airways of our stable COPD patients indicates a less-compromised picture than in peripheral lung compartments, where no significant variations were found for BMP4 immuno-expression in all lung compartments studied. 

We also found increased levels of the BMP4 inhibitory proteins BMPER, CRIM1, and chordin in the bronchial epithelium of COPD patients. The levels of CRIM1 and chordin were also increased in the bronchial lamina propria of COPD patients compared to CNS. Interestingly, the number of chordin+ cells/mm^2^ was significantly associated with the level of bronchial obstruction in COPD patients, suggesting a relationship between increased severity of the disease, BMP4 inhibition, and remodeling processes in the airways. Interestingly, we also found that treatment of 16HBE cells with IL-8—reported as increased in the bronchial biopsies of COPD patients [30]—significantly reduced BMP4 secretion. Macrophage-derived molecules, MIP1α and IL-27, upregulated the in vitro chordin levels in the 16HBE treated cells. These data suggest that the typical pro-neutrophil and macrophage pro-inflammatory processes occurring in the airways of COPD patients may drive the reduced expression of BMP4 and enhanced expression of the BMP4 inhibitor chordin in the bronchial epithelium. 

The noggin and chordin antagonists interact with BMPs to block the BMP ligand from binding to type I and type II receptors [31]. BMPER, in contrast, activates BMP4 at low concentrations but inhibits BMP4 signaling at higher concentrations in mouse endothelial cells [32]. Lung epithelial injury in vitro and in vivo disrupts epithelial barrier function, which is accompanied by increased BMP2 following epithelial damage, reduction in E-cadherin, and a decrease in BMPER expression [20,21]. High levels of BMPER antagonized BMP2-Smad5-Id1 signaling and restored epithelial integrity and homeostasis whilst BMP inhibition prevented the reduction in E-cadherin and disruption of epithelial barrier integrity and function [21]. BMPER is an extracellular matrix protein expressed by endothelial cells that modulates endothelial cell sprouting and migration by fine-tuning BMP4 activity during angiogenesis [33]. We found increased (albeit modestly expressed) BMPER levels in the bronchial epithelium of COPD patients compared to control non-smokers, suggesting that bronchial epithelial cells may contribute to maintaining epithelial barrier integrity and function by upregulating the BMPER protein in the large airways. In the peripheral lung compartments, our BMPER quantitation showed no significant differences between groups in all peripheral lung compartments studied.

CRIM1 is a glycosylated type I transmembrane protein expressed by a variety of adherent cells, including endothelial cells [34]. CRIM1 can interact with BMP4 and BMP7, acting as an antagonist when it is co-expressed with BMP4 and 7, reducing the production and secretion of mature BMP or tethering pre-BMP to the cell surface [13]. Even though it is only modestly expressed in the bronchial epithelium, CRIM1 was significantly increased in bronchial epithelial cells of our COPD patients compared to CNS; hence, it may act in COPD as an antagonist of the co-expressed BMP4. In the lamina propria, endothelial cells were the main site of CRIM1 expression and we observed a strong upregulation in COPD patients compared to CNS. In the peripheral lung compartments, our CRIM1 quantitation showed reduced levels in bronchiolar lamina propria and alveolar septa of COPD patients compared to control non-smokers. Transcriptomic data did not highlight any statistically significant trend but supported the considerable expression of this gene. 

Chordin functions have mainly been studied in the context of embryonic development and little is known about the role of chordin in chronic diseases such as COPD [9]. To the best of our knowledge, no data are available on chordin expression in the bronchi of COPD patients. BMP4 induces breast cancer migration, invasion, and metastasis, which is effectively blocked by chordin1 [10]. These authors also reported that high chordin1 mRNA levels in breast cancer patients were associated with longer disease and metastasis-free survival times than in patients with low-chordin1 mRNA-expressing tumors [10]. Similar results were also reported for lung cancer patients [10]. In the context of COPD, we observed a significant increase in chordin protein in the bronchial epithelium, particularly in SCOPD compared to CNS, and this increase was also seen in the lamina propria. Furthermore, chordin+ cells in the lamina propria were significantly correlated with the level of bronchial obstruction of our COPD patients. We speculate that chordin protein may play an important role in antagonizing BMP4, thereby preventing fibrosis in the large airways. On the contrary, in the peripheral lung compartments of COPD patients, our chordin quantitation showed it to be significantly decreased in alveolar macrophages compared to both control groups, i.e., smokers and non-smokers.

Transcriptomic data from bronchial rings did not show significant variations in mRNA expression of BMP molecules in MCOPD compared to CS and CNS, except for BMP1 and chordin, which were upregulated in CS compared to CNS, even though modestly expressed. These findings, only partially in line with the related proteins, suggest that post-transcriptional changes may occur. In the lung parenchyma, no significant differences were observed for any of the molecules studied. Interestingly, CRIM1 showed the highest mRNA expression levels, followed by BMP1, BMP2, and BMP4 (Table S1, Figure 5). As a limitation, we did not validate our transcriptomic data using qRT-PCR, although we adopted complement approaches to evaluate the corresponding protein levels (the effector molecules).

Since chordin has shown a protective effect by inhibiting the fibrosis processes and cardiomyocyte deaths [35], and given that inhibitors of BMP, mainly BMP4, promote epithelial and endothelial cell proliferation—as confirmed in our bronchial epithelial cells in vitro (Figure 5)—and given that exogenous BMP4 inhibits proliferation and differentiation in a clonal organoid tracheosphere assay [3,35,36,37], in light of these functions and considering our present immunohistochemical and in vitro results, we would argue that in the large airways, a more balanced picture of BMP4 and its antagonists is present, where increased chordin and CRIM1 may exert a protective function against fibrosis events or cell death. The parallel reduction in BMP4, particularly in more severe diseases, may favor the epithelial regeneration processes in this compartment of the airways. On the contrary, in the more compromised peripheral airways, BMP4 levels are unchanged in smokers and COPD patients vs. control non-smokers and the reduced levels of chordin and CRIM1 may exert a less protective effect against fibrosis events and pro-proliferative actions, particularly in alveolar septa (endothelial and epithelial components) and lung macrophages [3,35,36,37]. This imbalance in BMPs and BMP antagonists may play a role in the progressive reduction in their numbers in peripheral airways reported in patients with COPD [38,39,40,41], where this airway reduction becomes evident from the ninth airway generation to the peripheral lung [38,39,40,41]. In fact, these molecular alterations are also present, to a lesser extent, in smokers with near-normal lung function (see our present results in control smokers) and peripheral airway reduction has been reported as an early event in susceptible smokers for COPD development [39,40,41]. 

## 5. Conclusions

In conclusion, we have demonstrated an imbalance between the expression of BMP4 and that of BMP antagonists, CRIM1 and chordin, mainly in the peripheral airways and lung parenchyma of stable COPD patients. This imbalance may play a role in different biological processes such as induction of airway fibrosis, remodeling and impaired regenerative–reparative epithelial–endothelial responses. The clinical relevance of these observations needs to be further studied. Moreover, it is not known how current or new pharmacological treatments of COPD may influence this BMP4-antagonist molecular balance and its related effects in controlling the progression of the disease.

## Figures and Tables

**Table 1:** Clinical characteristics of subjects recruited for immunohistochemistry analysis of bronchial biopsies.

Groups	N	Age (Years)	M/F	Pack Years	Ex/Current Smokers	FEV_1_ (% Pred) Pre-β_2_	FEV_1_ (% Pred) Post-β_2_	FEV_1_/FVC (%)
**Control non-smokers**	11	64 ± 11	10/1	0	0	111 ± 16	ND	84 ± 9
**Control smokers with normal lung function**	13	60 ± 8	9/4	35 ± 25	2/11	101 ± 14	ND	81 ± 5
**COPD grades I and II (mild/moderate)**	18	68 ± 9	15/3	46 ± 22	6/12	66 ± 13 ^#^	70 ± 12	58 ± 9 ^#^
**COPD grades III and IV (severe/very severe)**	16	66 ± 12	11/5	58 ± 39	13/3	36 ± 7 ^#&^	41 ± 6	44 ± 9 ^#&^

Patients with chronic obstructive pulmonary disease (COPD) were classified according to GOLD 2011 (goldcopd.org) grades of severity using only the severity of airflow obstruction. For COPD patients, FEV_1_/FVC (%) are post-bronchodilator values. Abbreviations: M, male; F, female, FEV_1_: forced expiratory volume in 1 s; FVC, forced vital capacity; ND, not determined; Statistical analysis: ANOVA test ^#^: *p* < 0.0001, significantly different from control smokers with normal lung function and control non-smokers; ^&^: *p* < 0.0001, significantly different from mild/moderate COPD.

**Table 2:** Clinical characteristics of subjects recruited for immunohistochemistry analysis of the peripheral lung tissue.

Groups	N	Age (Year)	M/F	Ex/Current Smokers	Pack Years	FEV_1_ (% Pred) Pre-β_2_	FEV_1_ (% Pred) Post-β_2_	FEV_1_/FVC (%)
**Control non-smokers**	9	71 ± 3	5/4	---	---	115 ± 4.4	ND	81 ± 1.5
**Control smokers**	11	69 ± 1.9	7/4	7/4	39 ± 16	98 ± 2.3	ND	76 ± 1.6
**Patients with COPD**	9	70 ± 1.3	8/1	8/1	48 ± 26	69 ± 4.9 ^#^	77 ± 4.8	61 ± 2.9 ^#^

Patients with chronic obstructive pulmonary disease (COPD) were classified according to GOLD 2011 (goldcopd.org, 10 january, 2022) grades of severity using only the severity of airflow obstruction. For COPD patients, FEV_1_/FVC (%) are post-bronchodilator values. Abbreviations: M, male; F, female; FEV_1_, forced expiratory volume in 1 s; FVC, forced vital capacity; ND, not determined; Statistical analysis: ANOVA test ^#^: *p* < 0.0001, significantly different from control smokers with normal lung function and control non-smokers.

**Table 3:** Primary antibodies and immunohistochemical conditions used for the identification of BMPs and their antagonist proteins in bronchial biopsies.

Target	Supplier	Cat. #^a^	Source	Dilution	Positive Control
**BMP1**	R&D	AF1927	Goat	1:100 (2 µg/mL)	Nasal polyp
**BMP2**	Peprotech	500-p195	Rabbit	1:300 (0.3 µg/mL)	Nasal polyp
**BMP4**	Thermo Fisher	PA137175	Rabbit	1:150 (1.3 µg/mL)	Nasal polyp
**BMP7**	R&D	MAB3541	Mouse	1:300 (13 µg/mL)	Nasal polyp
**BMP9**	Abcam	Ab71809	Rabbit	1:80 (5 µg/mL)	Nasal polyp
**BMP10**	Bio Antibodies	Bs-9447R	Rabbit	1:150 (6.5 µg/mL)	Nasal polyp
**BMPER**	Abcam	Ab-73900	Rabbit	1:400 (1.2 µg/mL)	Nasal polyp
**CRIM1**	Sigma	HPA000556	Rabbit	1:25 (4 µg/mL)	Nasal polyp
**CHORDIN**	Cloud Clone	PAC126Hu01	Rabbit	1:80 (2.5 µg/mL)	Nasal polyp
**NOGGIN**	Santa Cruz	Sc-293439	Mouse	1:50 (2 µg/mL)	Nasal polyp

Cat. #^a^, catalogue.

**Table 4:** Immunohistochemical quantification of BMPs and BMP antagonists in bronchial biopsies.

	Control Non-Smokers	Control Smokers	Mild/Moderate COPD	Severe/Very Severe COPD	Kruskal–Wallis *p*-Value
**Epithelium (Score 0–3)**					
BMP1	0 (0–1)	0 (0–0.12)	0 (0–1.25)	0 (0–0.5)	0.957
BMP2	0 (0–0)	0 (0–0)	0 (0–0.12)	0 (0–0.12)	0.987
BMP4	0.75 (0.25–1.25)	0.5 (0.25–1.0) *	0.5 (0.25–0.75) *	0.37 (0.25–0.75) *	**0.010**
BMP7	0.25 (0.25–0.75)	0.25 (0–1.0)	0.5 (0.25–0.75)	0.5 (0.25–1.25)	0.264
BMP9	0.5 (0–1)	0.5 (0–1.25)	0.25 (0–1)	0.75 (0.25–1)	0.561
BMP10	0.5 (0–1.5)	0.75 (0–1)	0.62 (0.25–1.5)	0.75 (0.25–1.25)	0.745
BMPER	0 (0–0.25)	0.25 (0–0.75) *	0.5 (0–0.75) *	0.5 (0–1) *	**0.0009**
CRIM1	0 (0–1)	0.5 (0–1)	0.5 (0.25–1.5) *^&^	0.5 (0.25–1) *^$^	**0.0025**
CHORDIN	1.25 (0.5–2.0)	1.87 (0.5–2.75)	1.62 (1.25–2.5)	2 (1.5–3.0) *^$^	**0.0110**
NOGGIN	0 (0–0)	0 (0–0)	0 (0–0)	0 (0–0)	n.d.
**Lamina Propria (cells/mm^2^)**					
BMP1	6 (0–48)	4.5 (0–55)	12 (0–122)	9 (0–125)	0.765
BMP2	0 (0–0)	0 (0–0)	0 (0–12)	0 (0–64)	0.824
BMP4	76 (6–138)	48 (21–104)	69 (51–148)	84 (52–136)	0.054
BMP7	43 (5–142)	29 (9–116)	55 (24–123)	60 (20–97)	0.160
BMP9	48 (0–156)	27 (0–149)	14 (0–125)	55 (24–102)	0.109
BMP10	26 (0–122)	37 (6–90)	22 (0–106)	20 (0–52)	0.524
BMPER	89 (16–177)	150 (11–203)	150 (64–212)	135 (56–226)	0.062
CRIM1	5 (0–156)	113 (0–140)	124 (77–193) *^&^	140 (59–177) *^&^	**0.0003**
CHORDIN	175 (54–355)	226 (24–447)	145 (81–339)	384 (135–523) *^&$^	**0.0028**
NOGGIN	0 (0–11)	0 (0–8)	0 (0–6)	0 (0–6)	0.805

Data expressed as median (range); * significantly different from CNS; ^&^ significantly different from CS; ^$^ significantly different from mild/moderate COPD. n.d. = not determined; BMP = bone morphogenetic protein; BMPER = BMP endothelial cell precursor-derived regulator; CRIM1 = cysteine-rich motor neuron-1.

**Table 5:** Immunohistochemical quantification of BMP4 and related antagonists in the peripheral lung.

Localization	Control Non-Smokers	Control Smokers	COPD Patients	Kruskal–Wallis (*p*-Value)
Bronchiolar Epithelium (Score 0–3)				
BMP4	1.70 (0.5–2.5)	1.75 (1.4–2.5)	2.06 (0.5–2.5)	0.811
Chordin	1.75 (0.75–2.5)	1.50 (0.5–2.5)	1.75 (0.87–2.0)	0.495
CRIM1	0.62 (0.12–1.5) *	0.25 (0.12–1.0)	0.50 (0.12–1.5)	0.069
BMPER	0.25 (0–0.50)	0.25 (0.12–0.50)	0.50 (0.12–0.75)	0.593
Bronchiolar Lamina propria (Score 0–3)				
BMP4	0.50 (0.25–0.50)	0.50 (0.25–0.50)	0.50 (0–0.50)	0.931
Chordin	1.12 (0.25–1.5) *	0.50 (0.25–1.5)	0.75 (0.5–1.5)	0.054
CRIM1	0.62 (0.12–1.5) *	0.25 (0–0.75)	0.37 (0–1.5)	**0.030**
BMPER	0.50 (0.12–1.0)	0.50 (0.12–0.75)	0.75 (0.12–1.0)	0.371
Alveolar Macrophages (Score 0–3)				
BMP4	1.25 (1–2.5)	2.0 (1–2.5)	1.50 (1–2.0)	0.172
Chordin	2.0 (1–2.5) *	1.50 (1–2.0)	1.12 (0.5–2.5) &	**0.032**
CRIM1	0.87 (0.75–2.5)	0.75 (0.5–1.5)	0.87 (0.25–1.5)	0.720
BMPER	0.75 (0.25–1.5)	0.75 (0.25–1.25)	0.5 (0.25–1.5)	0.128
Alveolar Septa (Score 0–3)				
BMP4	0.25 (0.25–0.5)	0.37 (0.25–0.5)	0.5 (0.25–1.0)	0.128
Chordin	1.50 (0.75–2.25)	1.0 (0.5–2.0)	0.87 (0.5–1.5) &	0.087
CRIM1	1.37 (0.25–2.0) *	0.50 (0–1.0)	0.50 (0–2.0)	**0.010**
BMPER	0.75 (0.25–1.0)	0.50 (0.12–0.75)	0.25 (0.12–1.0)	0.287
Lung vessels (Score 0–3)				
BMP4	0.62 (0.25–1.5)	0.87 (0.25–1.0)	0.75 (0–1.5)	0.978
Chordin	1.37 (0.5–2.0)	0.75 (0–2.0)	0.50 (0–1.5)	0.318
CRIM1	1.50 (0.75–2.5)	1.5 (0.25–1.5)	1.25 (0–2.5)	0.426
BMPER	0.75 (0.25–1.0)	0.37 (0–1.0)	0.5 (0.25–1.0)	0.561

Mann–Whitney U test: * *p* < 0.05 from control smokers; & *p* < 0.05 from control non-smokers. Data expressed as median (range). In bold are significant data.

## Data Availability

Data are available on request from the authors.

## Supplementary Materials

The following supporting information can be downloaded at: https://www.mdpi.com/article/10.3390/biology12101304/s1. References [24,25,27] are cited in the Supplementary Materials. Table S1: Gene expression levels of the selected genes. For each gene, the expression level in the bronchial rings and lung parenchyma of control non-smokers, control smokers and COPD patients are reported as Transcript Per Millions (TPMs) together with fold-change and *p*-value for the different comparisons. Statistically significant comparisons are in bold.
