# Supplementary material for: Bone Morphogenic Proteins and Their Antagonists in the Lower Airways of Stable COPD Patients
Source: Biology (Basel). 2023 Oct 3;12(10):1304. doi: 10.3390/biology12101304 (PMC10603834; doi:10.3390/biology12101304)
Supplement: Supplementary file 1 [file biology-12-01304-s001.zip › biology-2575531-supplementary.pdf]

Supplementary Material

Lung function tests and volumes

Lung function tests included measurements of FEV_1 and FEV_1/FVC under baseline conditions in all the subjects examined (6200 Autobox Pulmonary Function Laboratory; Sensormedics Corp., Yorba Linda, CA, USA). In subjects with $FEV_1/FVC\% \leq 70\%$ pre-bronchodilator, the FEV_1 and $FEV_1/FVC\%$ measurements were repeated 20 min after the inhalation of 0.4 mg of salbutamol in order to assess the reversibility of airflow obstruction and post bronchodilator functional values.

Fiberoptic Bronchoscopy, Collection and Processing of Bronchial Biopsies

Subjects attended the bronchoscopy suite at 8.30 AM after having fasted from midnight and were pre-treated with atropine (0.6 mg IV) and midazolam (5-10 mg IV). Oxygen (3 l/min) was administered via nasal prongs throughout the procedure and oxygen saturation was monitored with a digital oximeter. Using local anesthesia with lidocaine (4%) to the upper airways and larynx, a fiberoptic bronchoscope (Olympus BF10 Key-Med, Southend, UK) was passed through the nasal passages into the trachea. Further lidocaine (2%) was sprayed into the lower airways, and four bronchial biopsy specimens were taken from segmental and subsegmental airways (4th to 6th airway generation) of the right lower and upper lobes using size 19 cupped forceps. Bronchial biopsies for immunohistochemistry were gently extracted from the forceps and processed for light microscopy [1]. At least two samples were embedded in Tissue Tek II OCT (Miles Scientific, Naperville, IL, USA), frozen within 15 min in isopentane precooled in liquid nitrogen, and stored at –80°C. The best frozen sample was then oriented and 6μm thick cryostat sections were cut for immunohistochemical staining and processed as described below.

Collection and Processing of the Peripheral Lung Tissue

Twenty-nine subjects undergoing lung resection surgery for a solitary peripheral neoplasm were recruited. Nine were non-smokers with normal lung function, 11 were smokers with normal lung function and 9 were smokers with COPD (**Table 2**). All former smokers had stopped smoking for more than one year. No subject had undergone preoperative chemo- or radiotherapy nor been treated with bronchodilators, theophylline, antibiotics, antioxidants or glucocorticoids in the month prior to surgery. Lung tissue processing was performed as previously described [1,2]. Two randomly selected tissue blocks and one bronchial ring were taken from the lungs obtained at surgery, avoiding areas grossly invaded by tumor. Samples were frozen in liquid nitrogen and stored at -80C. Serial sections 6 μm thick from frozen specimens were first cut and stained with hematoxylin-eosin (H&E) in order to visualize the morphology and to exclude the presence of microscopically evident tumor infiltration. Specimens were then cut for immunohistochemical staining and placed on charged slides as previously reported [1,2].

Cell culture and treatments

We used the SV40 large T antigen-transformed 16HBE cell line which retains the differentiated morphology and function of normal human bronchial epithelial cells (NHBE) [3]. 16HBE cells were maintained in Dulbecco's modified minimum essential medium (DMEM), supplemented with 10% v/v fetal bovine serum (FBS), 50 IU/ml penicillin, 50 μg/ml streptomycin, 1x non-essential amino acids, 1mM sodium pyruvate and 2mM glutamine (37°C, 5% CO_2) [3]. When cells were 60-70% confluent, the complete medium was replaced with DMEM

with 1% FBS for starvation time (24 h). 16HBE cells were cultured for 0-24 h because of their lower resistance to starvation. Non-treated 16HBE cells were used as controls. All experiments were performed in quadruplicate, i.e. four independent experiments for each type of treatment (BMP4 10 and 50 ng/ml, BMP4+LDN-193189) and time exposure (2 h).

Statistical analysis applied to functional and morphological data

Group data were expressed as mean (standard deviation) for functional data or median (range) or interquartile range (IQR) for morphologic data. Differences between groups were analyzed using analysis of variance (ANOVA) for functional data. ANOVA was followed by an unpaired t-test for comparison between groups. The Kruskal Wallis test was applied to the morphologic data followed by a Mann-Whitney U-test for comparison between groups. *In vitro* data were analyzed by the Mann-Whitney U test. Correlation coefficients were calculated using the Spearman rank method. Probability values of $p<0.05$ were considered significant. Data analysis was performed using the Stat View SE Graphics program (Abacus Concepts Inc., Berkeley, CA, USA).

Table S1: Gene expression levels of the selected genes. For each gene, the expression level in the bronchial rings and lung parenchyma of control non-smokers, control smokers and COPD patients are reported as Transcript Per Millions (TPMs) together with fold-change and p-value for the different comparisons. Statistically significant comparisons are in bold.

Bronchial Rings

Gene ID	CS vs. COPD - Fold change	CS vs. COPD - P-value	CNS vs. COPD - Fold change	CNS vs. COPD - P-value	CNS vs. CS - Fold change	CNS vs. CS - P-value	CNS - Mean	COPD - Mean	CS - Mean
BMP1	1.54	0.15	**-2.22**	**0.01**	**-3.40**	**0.00**	1.15	5.28	8.41
BMP2	-1.11	0.71	1.00	0.99	1.11	0.71	3.20	5.59	6.52
BMP4	-1.38	0.21	1.17	0.56	1.61	0.07	6.33	9.38	9.16
BMP7	-1.24	0.62	-1.16	0.73	1.07	0.88	2.52	5.06	4.67
BMP9	-9.47	0.09	-5.69	0.21	1.66	0.77	0.00	0.03	0.00
BMP10	na	na	na	na	na	na	0.00	0.00	0.00
BMPER	2.02	0.11	1.91	0.15	-1.06	0.89	0.88	0.98	2.19
CRIM1	1.37	0.28	-1.28	0.39	-1.76	0.05	11.08	27.61	41.42
CHRD	1.46	0.30	-1.94	0.07	**-2.83**	**0.00**	0.54	2.35	3.58
NOG	3.87	0.11	2.41	0.31	-1.60	0.57	0.24	0.20	0.82

Lung Parenchyma

Gene ID	CNS vs. COPD - Fold change	CNS vs. COPD - P-value	CS vs. COPD - Fold change	CS vs. COPD - P-value	CS vs. CNS - Fold change	CS vs. CNS - P-value	CNS - Mean	COPD - Mean	CS - Mean
BMP1	-1.17	0.53	1.22	0.42	1.43	0.16	25.77	30.99	39.25
BMP2	1.01	0.94	1.00	0.98	-1.01	0.96	32.29	32.55	33.38

BMP4	1.52	0.02	1.39	0.06	-1.09	0.62	18.05	11.94	16.90
BMP7	-1.28	0.54	-1.25	0.56	1.02	0.96	2.86	3.67	2.95
BMP9	na	na	na	na	na	na	0.00	0.00	0.00
BMP10	na	na	na	na	na	na	0.00	0.00	0.00
BMPER	1.12	0.70	1.10	0.75	-1.02	0.95	4.33	4.02	4.32
CRIM1	1.36	0.08	1.18	0.32	-1.15	0.42	217.97	163.35	193.95
CHRD	1.08	0.76	-1.28	0.30	-1.39	0.19	5.82	5.72	4.27
NOG	1.32	0.46	1.02	0.96	-1.30	0.49	2.65	1.93	2.05

CS: control smokers; CNS: control non smokers; COPD: chronic obstructive pulmonary disease

1. Di Stefano, A., Caramori, G., Barczyk, A., Vicari, C., Brun, P., Zanini, A., Cappello, F., Garofano, E., Padovani, A., Contoli, M., Casolari, P., Durham, A.L., Chung, K.F., Barnes, P.J., Papi, A., Adcock, I., Balbi, B. Innate immunity but not NLRP3 inflammasome activation correlates with severity of stable COPD. Thorax. 2014; 69:516-24.
2. Di Stefano, A., Sangiorgi, C., Gnemmi, I., Casolari, P., Brun, P., Ricciardolo, F.L.M., Contoli, M., Papi, A., Maniscalco, P., Ruggeri, P., Girbino, G., Cappello, F., Pavlides, S., Guo, Y., Chung, K.F., Barnes, P.J., Adcock, I.M., Balbi, B., Caramori, G. TGF-β Signaling Pathways in Different Compartments of the Lower Airways of Patients With Stable COPD. Chest. 2018; 153:851-862.
3. Cozens, A.L., Yezzi, M.J., Kunzelmann, K., Ohrui, T., Chin, L., Eng, K., Finkbeiner, W.E., Widdicombe, J.H., Gruenert, D.C. CFTR expression and chloride secretion in polarized immortal human bronchial epithelial cells. Am J Respir Cell Mol Biol. 1994;10:38-47.
